# Carbonylation Modification Regulates Na/K-ATPase Signaling and Salt Sensitivity: A Review and a Hypothesis

**DOI:** 10.3389/fphys.2016.00256

**Published:** 2016-06-28

**Authors:** Preeya T. Shah, Rebecca Martin, Yanling Yan, Joseph I. Shapiro, Jiang Liu

**Affiliations:** Department of Pharmacology, Physiology and Toxicology, Joan C. Edwards School of Medicine, Marshall UniversityHuntington, WV, USA

**Keywords:** Na/K-ATPase, ROS, protein carbonylation, signaling

## Abstract

Na/K-ATPase signaling has been implicated in different physiological and pathophysiological conditions. Accumulating evidence indicates that oxidative stress not only regulates the Na/K-ATPase enzymatic activity, but also regulates its signaling and other functions. While cardiotonic steroids (CTS)-induced increase in reactive oxygen species (ROS) generation is an intermediate step in CTS-mediated Na/K-ATPase signaling, increase in ROS alone also stimulates Na/K-ATPase signaling. Based on literature and our observations, we hypothesize that ROS have biphasic effects on Na/K-ATPase signaling, transcellular sodium transport, and urinary sodium excretion. Oxidative modulation, in particular site specific carbonylation of the Na/K-ATPase α1 subunit, is a critical step in proximal tubular Na/K-ATPase signaling and decreased transcellular sodium transport leading to increases in urinary sodium excretion. However, once this system is overstimulated, the signaling, and associated changes in sodium excretion are blunted. This review aims to evaluate ROS-mediated carbonylation of the Na/K-ATPase, and its potential role in the regulation of pump signaling and sodium reabsorption in the renal proximal tubule (RPT).

Accumulating evidence suggests that excessive dietary salt intake may play a role in the pathogenesis of hypertension, with more pronounced effects seen in salt-sensitive patients (Calhoun et al., 2008). Consequently, modest restriction of dietary salt and diuretic therapy are often recommended for treatment of resistant hypertension, particularly within the salt-sensitive sub-group (He and MacGregor, 2004; Calhoun et al., 2008). Long-term blood pressure (BP) regulation is highly associated with renal sodium handling (Guyton, 1991). Recent studies observe the CTS-activated Na/K-ATPase signaling pathway to contribute to RPT sodium handling and salt sensitivity (Buckalew, 2005; Meneton et al., 2005; Schoner and Scheiner-Bobis, 2007, 2008; Bagrov and Shapiro, 2008; Fedorova et al., 2010; Liu and Xie, 2010). Various intercellular and extracellular functions are regulated by the signaling function of the Na/K-ATPase. Discussion of Na/K-ATPase signaling and the downstream physiological and pathophysiological implications can be found in several references (Bertorello and Sznajder, 2005; Buckalew, 2005; Aperia, 2007; Liu and Shapiro, 2007; Schoner and Scheiner-Bobis, 2007, 2008; Bagrov and Shapiro, 2008; Bagrov et al., 2009; Blaustein et al., 2009; Li and Xie, 2009; Fedorova et al., 2010; Liu and Xie, 2010). Based on our recent observations, we focus on the effect of oxidative (carbonylation) modification of Na/K-ATPase and sodium handling in RPTs.

## Protein carbonylation and cell signaling

Biologically, electron reduction of oxygen (O_2_) leads to generation of ROS including superoxide (${\text{O}}_{2}^{–}$), hydrogen peroxide (H_2_O_2_), and hydroxyl radical (HO^›^). ROS is able to oxidize various types of biological molecules including proteins, lipids, and DNA, leading to their functional changes. Through Fenton's reaction, H_2_O_2_ (generated by ${\text{O}}_{2}^{–}$ or via other mechanisms) is reduced to HO^›^ by coupling oxidation of reduced ferrous ion (Fe^2+^) to ferric ion (Fe^3+^). This metal-catalyzed oxidation (MCO) process oxidizes proteins by introducing carbonyl groups (such as aldehydes, ketones, or lactams) into the side chains of certain amino acids (such as proline, arginine, lysine and threonine; Stadtman and Berlett, 1991; Stadtman and Levine, 2000; Nyström, 2005). Unlike this direct (primary) carbonylation, indirect (secondary) carbonylation on lysine, cysteine, and histidine can occur by reactive carbonyl compounds generated from other types of oxidation, such as lipid and carbohydrate oxidation via Michael addition reactions and formation of Schiff bases. Protein carbonylation is a well-recognized marker of oxidative stress because of its stability, its effect on protein functions, and its link to various biological and pathological conditions. Oxidative stress has been implicated in the aging process, various conditions like ischemia-reperfusion and hyperoxia, and various human diseases like Alzheimer's disease, chronic lung disease, chronic renal failure, diabetes, and sepsis (Stadtman and Levine, 2000; Dalle-Donne et al., 2003, 2006a,b). Since the Fenton reaction involves the conversion of H_2_O_2_ to HO^•^, any specie of ROS with H_2_O_2_ as an intermediate and/or end product may stimulate the reaction. In biological systems, H_2_O_2_ is one of the most common end products of most ROS generating systems.

Oxidative modification of protein, reversible and irreversible, dynamically regulates protein structure, function, and trafficking, as well as cellular signaling and function (Go and Jones, 2013). Direct protein carbonylation is very stable and “chemically” irreversible (Stadtman and Berlett, 1991; Nyström, 2005). Recent studies from the Suzuki laboratory have demonstrated the role of the carbonylation/decarbonylation process in ROS signal transduction in which thiol groups were responsible for decarbonylation via enzymatic processes, likely through thioredoxin reductase (Wong et al., 2008, 2010, 2012, 2013).

## CTS, the Na/K-ATPase, and renal sodium handling

CTS, also known as endogenous digitalis-like substances, include plant-derived glycosides and vertebrate-derived aglycones (Schoner and Scheiner-Bobis, 2007, 2008). Although, the production and secretion of endogenous CTS are not completely understood, they appear to be regulated by angiotensin II and adrenocorticotropic hormone (Hamlyn et al., 1991; Laredo et al., 1997; Schoner and Scheiner-Bobis, 2007; Bagrov et al., 2009). CTS are present in measurable amounts under normal physiological conditions, and are elevated under a number of pathological states. Different species of endogenous CTS show variations in kinetics and tissue action in response to salt loading in both animal models and in human hypertensive patients (Haddy and Pamnani, 1998; Fedorova et al., 2005; Manunta et al., 2006; Schoner and Scheiner-Bobis, 2007, 2008).

The Na/K-ATPase belongs to the P-type ATPase family and consists of two non-covalently linked α and β subunits. Several α and β isoforms, expressed in a tissue-specific manner, have been identified and functionally characterized (Sweadner, 1989; Blanco and Mercer, 1998; Kaplan, 2002; Sanchez et al., 2006). The α1 subunit contains multiple structural motifs that interact with soluble, membrane and structural proteins (Jordan et al., 1995; Beggah and Geering, 1997; Feschenko et al., 1997; Zhang et al., 1998, 2006a; Yudowski et al., 2000; Lee et al., 2001; Xie and Cai, 2003; Barwe et al., 2005; Song et al., 2006; Tian et al., 2006). Binding to these proteins not only regulates the ion pumping function of the enzyme, but it also conveys signal transducing functions to the Na/K-ATPase (Xie and Cai, 2003; Kaplan, 2005; Kaunitz, 2006; Schoner and Scheiner-Bobis, 2007; Li and Xie, 2009).

It has been hypothesized for years that increases in endogenous CTS enhance natriuresis and diuresis by direct inhibition of renal tubular Na/K-ATPase, leading to reduced renal reabsorption of filtered sodium (Blaustein, 1977; Haddy et al., 1979; de Wardener and Clarkson, 1985). The first unequivocal demonstration of ouabain-like substance in human plasma was reported 25 years ago (Hamlyn et al., 1991). *In vivo* experiments suggest the essential role of endogenous CTS in modulating renal sodium excretion and BP with different approaches. First, administration of some (e.g., ouabain) but not all CTS induces natriuresis (Foulkes et al., 1992; Yates and McDougall, 1995). Second, in transgenic mice expressing ouabain-sensitive Na/K-ATPase α1 subunit, both acute salt load and ouabain infusion augment natriuretic responses, which may be inhibited by administration of an anti-digoxin antibody fragment (Dostanic-Larson et al., 2005; Loreaux et al., 2008). Third, immune-neutralization of endogenous CTS prevents CTS mediated natriuretic and vasoconstrictor effects (Fedorova et al., 2001, 2002; Bagrov and Shapiro, 2008; Nesher et al., 2009). Fourth, administration of the ouabain-antagonist, rostafuroxin (previously PST 2238) not only prevents ouabain induced Na/K-ATPase signaling, but also prevents ouabain-induced increase in BP (Ferrandi et al., 2004). Finally, in humans, high salt intake increases circulating endogenous CTS (Manunta et al., 2006; Anderson et al., 2008; Bagrov and Shapiro, 2008). Increased CTS excretion is directly linked to enhanced RPT-mediated fractional Na+ excretion, but inversely related to age and to age-dependent increase in salt-sensitivity (Anderson et al., 2008).

## The Na/K-ATPase signaling and salt sensitivity

Although historical focus has largely been on the direct inhibition of the Na/K-ATPase ion-exchange activity and sodium reabsorption in RPTs by CTS, this does not appear to be the predominant mechanism for several reasons. In contrast, the newly appreciated signaling function of Na/K-ATPase has been widely confirmed and provides a realistic, mechanistic framework that we will discuss further. We have observed that the renal Na/K-ATPase and its signaling play a key role in regulating renal sodium handling (Liu et al., 2002, 2004, 2005, 2011; Periyasamy et al., 2005; Oweis et al., 2006; Cai et al., 2008; Yan et al., 2013).

**Figure 1:**
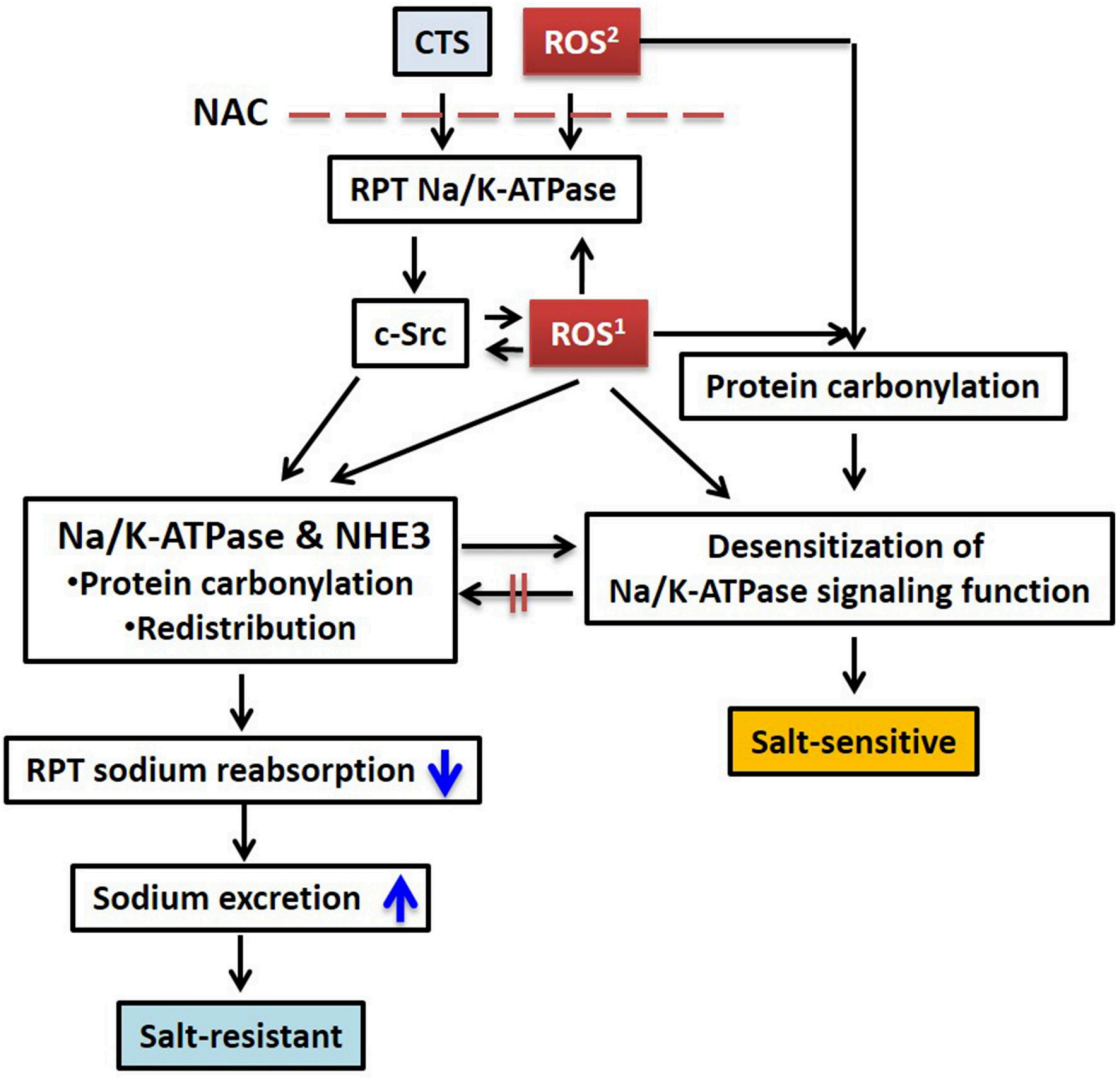
**Schematic illustration of CTS and ROS mediated sodium handling in RPT and its relation to salt sensitivity**. CTS induced intracellular ROS generation and extracellularly generated ROS stimulates Na/K-ATPase/c-Src signaling, protein carbonylation, and transporter redistribution. Pretreatment with NAC blocks this process. ROS1, CTS induced intracellular ROS generation through Na/K-ATPase signaling; ROS2, extracellular ROS generated by glucose oxidase or other stimuli. Please also see **Figure 2**. RPT, renal proximal tubule; NAC, N-acetyl-L-cysteine.

Decreases in basolateral Na/K-ATPase activity alone do not appear sufficient to decrease net sodium reabsorption across the renal tubular epithelium. In porcine RPT LLC-PK1 cells, ouabain activates the Na/K-ATPase signaling pathways and consequently redistributes the basolateral Na/K-ATPase and the apical sodium/hydrogen exchanger isoform 3 (NHE3) in a coordinated manner; this leads to symmetrical reduction of cell surface Na/K-ATPase and NHE3 expression, and ultimately decreases net transcellular sodium transport (Liu et al., 2002, 2004, 2005; Oweis et al., 2006; Cai et al., 2008; Figure 1). In this experimental model, the concentrations of ouabain used *in vitro* were chosen to mimic the concentrations of CTS seen *in vivo* with salt loading. No significant acute change in intracellular Na^+^ concentration was observed (Cai et al., 2008), further suggesting the coordination of the downregulation of both apical and basolateral sodium transporters. This Na/K-ATPase signaling mediated regulation of renal tubular epithelial ion transporters was additionally confirmed in *in vivo* studies (Periyasamy et al., 2005; Liu et al., 2011).

The Dahl salt-resistant (R) and salt-sensitive (S) strains were developed from Sprague Dawley rat strain by selective breeding, depending on the resistance or susceptibility to the hypertensive effects of high dietary sodium (Dahl et al., 1962). In these strains, sodium handling within the RPT is an essential determinant of their different BP responses (Dahl et al., 1974; Rapp, 1982; Rapp and Dene, 1985; Mokry and Cuppen, 2008). At the cost of elevated systolic BP, Dahl S rats rid excess sodium primarily via pressure-natriuresis. In contrast, Dahl R rats counterbalance salt loading via significant reduction of renal sodium reabsorption without increasing BP. *In vivo* studies indicate that impaired RPT Na/K-ATPase signaling appears to be causative of experimental Dahl salt-sensitivity (Liu et al., 2011). Specifically, in Dahl R rats (Jr strain), a high salt diet (2% NaCl for 7 days) and exposure to ouabain activates RPT Na/K-ATPase signaling and stimulates coordinated redistribution of Na/K-ATPase and NHE3, resulting with increases in renal sodium excretion. However, this does not occur in age- and gender-matched Dahl S rats (Jr strain; Liu et al., 2011). At present, we do not have a simple explanation for this occurrence. First, the α1 subunit is essentially the only α isoform expressed in RPTs (Blanco and Mercer, 1998; Summa et al., 2004) and genes coding α1 subunit and NHE3 (in rat chromosomes 1 and 2, respectively) are not located in identified and proposed BP quantitative trait loci (Joe, 2006). Second, there is no difference in α1 gene (Atp1a1) coding (Mokry and Cuppen, 2008), α1 ouabain-sensitivity (Nishi et al., 1993), and α1 expression (Liu et al., 2011) between these two strains. Third, acute salt-loading increases circulating CTS (ouabain and MBG) in both S and R rats (Fedorova et al., 2000). These observations suggest that there must be resistance to CTS signaling in the Dahl S rat, a phenomenon that we only partially understand and will discuss further below.

## ROS and the Na/K-ATPase signaling

It is well established that an increase in oxidative stress occurs in many forms of experimental hypertension (Kitiyakara et al., 2003; Touyz, 2004; Wilcox, 2005; Vaziri and Rodriguez-Iturbe, 2006; Welch, 2006). We and others have observed that a high salt diet stimulates endogenous CTS release and ROS generation within the RPT (Moe et al., 1991; Yang et al., 2008; Panico et al., 2009; McDonough, 2010; Banday and Lokhandwala, 2011; Liu et al., 2011). The increases in ROS (Meng et al., 2002; Kitiyakara et al., 2003; Taylor et al., 2006) regulate physiological processes including renal tubular ion transport, fluid reabsorption, and sodium excretion (Moe et al., 1991; Zhang et al., 2002; Garvin and Ortiz, 2003; Han et al., 2005; Yang et al., 2008; Panico et al., 2009; Wang et al., 2009; Banday and Lokhandwala, 2011; Liu et al., 2011; Schreck and O'Connor, 2011). In particular, increases in ROS regulate the activity and cellular distribution of the basolateral Na/K-ATPase as well as the apical NHE3 and sodium/glucose cotransporter, at least under normal circumstances (Moe et al., 1991; Fisher et al., 2001; Silva and Soares-da-Silva, 2007; Yang et al., 2007, 2008; Panico et al., 2009; Crajoinas et al., 2010; Johns et al., 2010; Liu et al., 2011). In our *in vitro* studies with LLC-PK1 cells, we have observed that ouabain stimulates generation of ROS which is critical in CTS-mediated Na/K-ATPase signaling, transporter trafficking, and ^22^Na^+^ flux (Yan et al., 2013). Pre-treatment with higher doses, but not a low dose, of anti-oxidant N-acetyl-L-cysteine (NAC) attenuated the effect of ouabain on c-Src activation and transcellular ^22^Na^+^ flux, suggesting a role of basal physiological redox status in the initiation of ouabain induced Na/K-ATPase signaling. This is analogous to the observation that the Na/K-ATPase activity is redox-sensitive with an “optimal redox potential range” (Petrushanko et al., 2006). While CTS stimulates ROS generation and Na/K-ATPase signaling in different *in vitro* and *in vivo* models (Xie et al., 1999; Liu et al., 2000, 2006; Tian et al., 2003; Kennedy et al., 2006a,b; Elkareh et al., 2007), glucose oxidase-induced H_2_O_2_ alone also stimulates Na/K-ATPase signaling, promotes Na/K-ATPase endocytosis, and inhibits active transcellular ^22^Na^+^ transport (Liu et al., 2006; Yan et al., 2013). The phenomenon of redox-sensitivity of the Na/K-ATPase has been demonstrated within many animal species, tissues, and cell types. Oxidative modification can affect Na/K-ATPase activity through different mechanisms. For example, S-glutathionylation is the formation of a mixed disulphide (cysteine-S-S-glutathione) between cysteine-SH with glutathione-SH or thiol-disulfide exchange. S-glutathionylation of cysteine residue(s) of the Na/K-ATPase α subunit can block the intracellular ATP-binding site, leading to inhibition of its enzymatic activity (Petrushanko et al., 2012). Ouabain-induced S-glutathionylation of cysteine of the Na/K-ATPase β1 subunit, a process affected by Na/K-ATPase conformational poise (Liu et al., 2012), reduces α1/β1 association and enzymatic activity by stabilizing the enzyme in an E2-prone conformation (Figtree et al., 2009). Oxidants and oxidative modification of the Na/K-ATPase can lead to functional changes (Kim and Akera, 1987; Xie et al., 1990; Huang et al., 1992; Mense et al., 1997; Thevenod and Friedmann, 1999; Zhang et al., 2002; Ellis et al., 2003; Bogdanova et al., 2006; Liu et al., 2006; Reifenberger et al., 2008; Blaustein et al., 2009; Figtree et al., 2009; White et al., 2009; Bibert et al., 2011; Figtree et al., 2012; Petrushanko et al., 2012; Soares-da-Silva, 2012) and formation of Na/K-ATPase oligomeric structure (Dobrota et al., 1999). As partner of Na/K-ATPase signaling, tyrosine kinase c-Src and lipid rafts (including caveolae structural component caveolins) are also redox-sensitive and critical in redox signaling platform formation (Seshiah et al., 2002; Zuo et al., 2005; Touyz, 2006; Zhang et al., 2006b; Han et al., 2008). This suggests a redox-sensitive Na/K-ATPase signaling and its possible role in ROS regulation.

Both ouabain and glucose oxidase-induced H2O2 stimulate Na/K-ATPase signaling and neutralization of the increase in ROS attenuated ouabain-induced effects (Xie et al., 1999; Liu et al., 2000, 2006; Tian et al., 2003; Kennedy et al., 2006a; Elkareh et al., 2007; Yan et al., 2013; Wang et al., 2014). We further observed that both ouabain and glucose oxidase-induced H2O2 stimulate direct protein carbonylation of Pro222 and Thr224 residues of the Na/K-ATPase α1 subunit (α1 carbonylation) in LLC-PK1 cells (Yan et al., 2013). The Pro222 and Thr224 are located in peptide 211VDNSSLTGESEPQTR225 [UniProtKB/Swiss-Prot No P05024 (AT1A1_PIG)]. While the α1 subunit is highly conserved amongst human, pig, rat, and mouse (the homology is over 98.5%), the identified peptide is 100% identical amongst these four species (Table 1). This peptide is located in the actuator (A) domain of α1 subunit, and Pro222/Thr224 are highly exposed and facing the nucleotide binding (N) domain of the α1 subunit. Upon ouabain binding, Na/K-ATPase undergoes conformational changes, in which the A domain is rotated to the N domain. Structure-function analysis indicates that these conformational changes may affect binding of the α1 subunit to signaling molecules such as c-Src and PI3K (Yatime et al., 2011). In addition, the peptide also contains the TGES motif that is the anchor of A domain rotation (Yatime et al., 2011). In immunoprecipitated α1 subunit, both ouabain and glucose oxidase do not induce formation of advanced glycation end products (AGEs) adducts. Like ouabain, glucose oxidase is able to activate Na/K-ATPase signaling, leading to reduction of transcellular^22^Na^+^ transport.

**Table 1:** **Partial alignment of α1 subunit of human, pig, rat, and mouse**.

**SP|P05023|ATA1_HUMAN**	**211**	**CKVDNSSLTGESE${\text{P}}^{224}$Q${\text{T}}^{226}$RSPDFTNENPLETR**	**240**
**SP|P05024|ATA1_PIG**	**209**	**CKVDNSSLTGESE${\text{P}}^{222}$Q${\text{T}}^{224}$RSPDFTNENPLETR**	**238**
**SP|P06685|ATA1_RAT**	**211**	**CKVDNSSLTGESE${\text{P}}^{224}$Q${\text{T}}^{226}$RSPDFTNENPLETR**	**240**
**SP|Q8VDN2|ATA1_MOUSE**	**211**	**CKVDNSSLTGESE${\text{P}}^{224}$Q${\text{T}}^{226}$RSPDFTNENPLETR**	**240**

*Pro and Thr are shown in red*.

Recent studies suggest that, in biological systems, protein carbonylation is reversible (decarbonylation) and may function as regulatory mechanism of cell signaling (Wong et al., 2008, 2010, 2012, 2013). We also observed a decarbonylation mechanism, which apparently reverses the carbonylation of the Na/K-ATPase α1 subunit induced by CTS (Yan et al., 2013). Removal of ouabain from the culture medium clearly reverses ouabain-mediated carbonylation; inhibition of *de novo* protein synthesis as well as degradation pathways through lysosome and proteasome does not affect this decarbonylation, which is still poorly understood. It is possible that carbonylation modification might stabilize the Na/K-ATPase in a certain conformational status favoring ouabain binding to the Na/K-ATPase α1 subunit and ouabain-Na/K-ATPase signaling, as seen in S-glutathionylation of cysteine residue(s) of the Na/K-ATPase (Figtree et al., 2009; Petrushanko et al., 2012). Nevertheless, the underlying mechanism might be physiologically significant since the carbonylation/decarbonylation process could be an important regulator of the RPT Na/K-ATPase signaling and sodium handling.

## Oxidative (carbonylation) modification and salt sensitivity, a hypothesis

**Figure 2:**
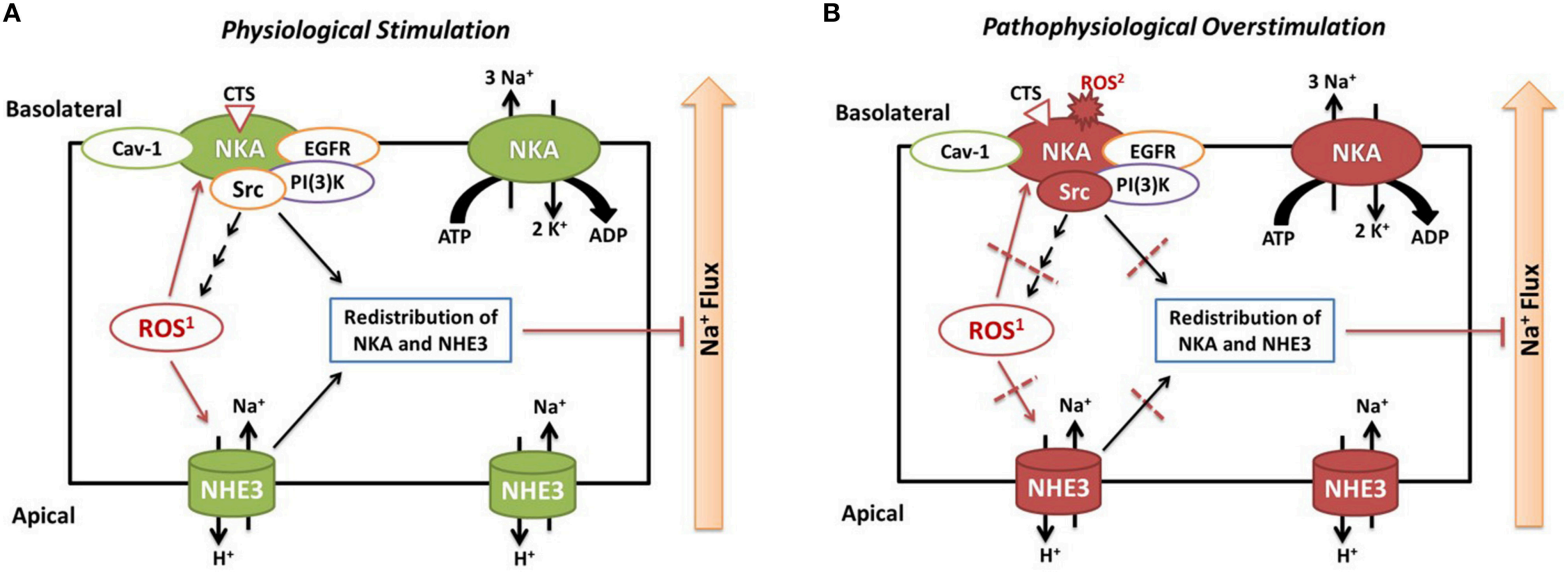
**Illustrated hypothesis of α1 carbonylation on Na/K-ATPase signaling and sodium handling**. Panel **(A)** shows events under physiological stimulation and Panel **(B)** shows events under pathophysiological overstimulation. ROS1, CTS induced intracellular ROS generation through Na/K-ATPase signaling; ROS2, extracellular ROS generated by glucose oxidase or other stimuli.

Based on our data and literatures, we propose that carbonylation modification of RPT Na/K-ATPase α1 subunit has biphasic effects. (1) Physiological and controllable α1 carbonylation stimulates Na/K-ATPase signaling and sodium excretion, rendering salt resistance (Figure 2A) whereas (2) prolonged exposure to oxidant stress leads to overstimulated α1 carbonylation and desensitized Na/K-ATPase signaling, effecting salt sensitivity (Figure 2B). First, Dahl S rats show considerably higher basal levels of oxidative stress than R rats, and high salt diets increase renal oxidative stresses that contribute to salt-sensitive hypertension (Meng et al., 2002; Kitiyakara et al., 2003; Taylor et al., 2006). Second, while high salt diets increase circulating CTS, we have observed that a high salt diet (HS, 2% NaCl for 7 days) stimulates the Na/K-ATPase signaling in isolated RPTs from Dahl R but not S rats (i.e., impaired Na/K-ATPase signaling in S rats; Liu et al., 2011). Third, in RPT LLC-PK1 cells, CTS- and H_2_O_2_-mediated redox-sensitive Na/K-ATPase signaling and α1 carbonylation is involved in this signaling process, in a feed-forwarding mechanism (Yan et al., 2013). Fourth, high but not low concentration of NAC is able to prevent α1 carbonylation and Na/K-ATPase signaling (Yan et al., 2013). Even though it is still not clear of the carbonylation/decarbonylation process, it is reasonable to postulate that prolonged excessive α1 carbonylation (by CTS and/or other factors) might overcome the decarbonylation capacity, leading to desensitization or termination of the Na/K-ATPase signaling function. This is reminiscent of the observations in clinical trials using antioxidant supplements. The beneficial effect of antioxidant supplements is controversial and not seen in most clinical trials with administration of antioxidant supplements (reviewed in Touyz, 2004; Munzel et al., 2010). Low doses of antioxidant supplementation may be ineffective, but high doses may be even dangerous since excess antioxidants might become pro-oxidants if they cannot promptly be reduced in the anti-oxidant chain (Huang et al., 2006). It appears that the balance of the redox status, within a physiological range, may be critical in order to maintain beneficial ROS signaling.

## Perspective

The Na/K-ATPase has recently emerged as a therapeutic target (Aperia, 2007; Yatime et al., 2009). A clearer understanding of the mechanisms whereby a CTS-ROS-Na/K-ATPase signaling axis counterbalancing salt retention would have major pathophysiological and therapeutic implications, and further explain the progressive impairment of renal sodium handling under excessive oxidative stresses such as hypertension, aging, obesity, and diabetes. Impairment of coordinated regulation of the basolateral Na/K-ATPase and the apical NHE3 antiporter is implicated in salt-sensitive BP changes. Furthermore, recent evidence suggests both the Na/K-ATPase and, its adjacent signaling counterpart, c-Src to be redox-sensitive. Although carbonylation modification of the Na/K-ATPase is involved in the Na/K-ATPase signaling, a more thorough mechanistic understanding is necessary. Some pertinent questions remain to be resolved, such as the possible effect of carbonylation on CTS binding affinity, Na/K-ATPase conformational change, mechanisms of carbonylation/decarbonylation, and the destiny of the carbonylated Na/K-ATPase.

## Author contributions

PS, RM, YY, JS, and JL discussed the topic and wrote the manuscript. YY, JS, and JL reviewed and commended on the manuscript. PS and RM did the final edit.

## Funding

This work was partially supported by NIH grants HL109015 and HL071556 to JS.

### Conflict of interest statement

The authors declare that the research was conducted in the absence of any commercial or financial relationships that could be construed as a potential conflict of interest.
